# Development and psychometric evaluation of the PMR-Impact Scale: a new patient reported outcome measure for polymyalgia rheumatica

**DOI:** 10.1093/rheumatology/keac317

**Published:** 2022-05-26

**Authors:** Helen Twohig, Caroline Mitchell, Christian D Mallen, Sara Muller

**Affiliations:** School of Medicine, Keele University, Keele; Academic Unit of Primary Medical Care, University of Sheffield, Sheffield, UK; School of Medicine, Keele University, Keele; School of Medicine, Keele University, Keele

**Keywords:** polymyalgia rheumatica, patient-reported outcomes, psychometrics

## Abstract

**Objectives:**

PMR causes pain, stiffness and disability in older adults. Measuring the impact of the condition from the patient’s perspective is vital to high-quality research and patient-centred care, yet there are no validated patient-reported outcome measures (PROMs) for PMR. We set out to develop and psychometrically evaluate a PMR-specific PROM.

**Methods:**

Two cross-sectional postal surveys of people with a confirmed diagnosis of PMR were used to provide data for field testing and psychometric evaluation. A total of 256 participants completed the draft PROM. Distribution of item responses was examined, and exploratory factor analysis and Rasch analysis were used to inform item reduction, formation of dimension structure and scoring system development. Some 179 participants completed the PROM at two time points, along with comparator questionnaires and anchor questions. Test–retest reliability, construct validity and responsiveness were evaluated.

**Results:**

Results from the field-testing study led to the formation of the PMR-Impact Scale (PMR-IS), comprising four domains (symptoms, function, psychological and emotional well-being, and steroid side effects). Construct validity and test–retest reliability met accepted quality criteria for each domain. There was insufficient evidence from this study to determine its ability to detect flares/deterioration, but the PMR-IS was responsive to improvements in the condition.

**Conclusion:**

The PMR-IS offers researchers a new way to assess patient-reported outcomes in clinical studies of PMR. It has been developed robustly, with patient input at every stage. It has good construct validity and test–retest reliability. Further work is needed to fully establish its responsiveness and interpretability parameters, and to assess its real-world clinical utility.

Rheumatology Key messages

- The PMR-Impact Scale (PMR-IS) assesses the impact of PMR from a patient perspective.
- It has good construct validity and test–retest reliability across all domains.
- This new patient-reported outcome measures offers a real opportunity to ensure future PMR research is patient-centred.

## Introduction

The lack of valid, reliable, patient-centred outcome measures hinders high quality research into PMR. PMR is an inflammatory musculoskeletal condition causing pain, stiffness and disability. Worldwide, it is most common in northern latitudes and populations of Scandinavian and Northern European descent [1]. In the UK, PMR is the most common inflammatory musculoskeletal condition presenting in older adults [2], with an overall incidence of 95.9 per 100 000 person years in those aged over 40 years, rising to 314.9 per 100 000 in the over 80s [3]. PMR can be challenging to diagnose and manage because of its heterogeneous presentation, variable disease course and the impact of comorbidities which are frequently present in this age group. Glucocorticoids remain the dominant treatment for the condition and the side effects of these drugs need to be balanced against control of symptoms.

Many questions remain about the optimal management of PMR. The 2015 EULAR/ACR PMR clinical guidelines [4] highlight the need to identify which outcome measures (including patient-related outcomes), and response, remission and relapse criteria should be used in people with PMR. Indeed, it could be argued that if progress is to be made with any of the items on the research agenda, it is essential to establish a way to measure the impact of the condition, and of its treatment, on the people it affects. A recent systematic review of outcomes measured in studies of PMR and the validity of instruments used [5] found that current measures are not patient-centred and that there is scant evidence on their measurement properties to support their use in PMR. This lack of psychometrically robust outcome measures limits the development of new therapeutic interventions for this patient group. We therefore set out to develop and evaluate the psychometric properties of a patient-reported outcome measure (PROM) to assess the impact of PMR on a person’s life, for use in clinical research: the PMR-Impact Scale (PMR-IS).

## Methods

**Fig. 1:**
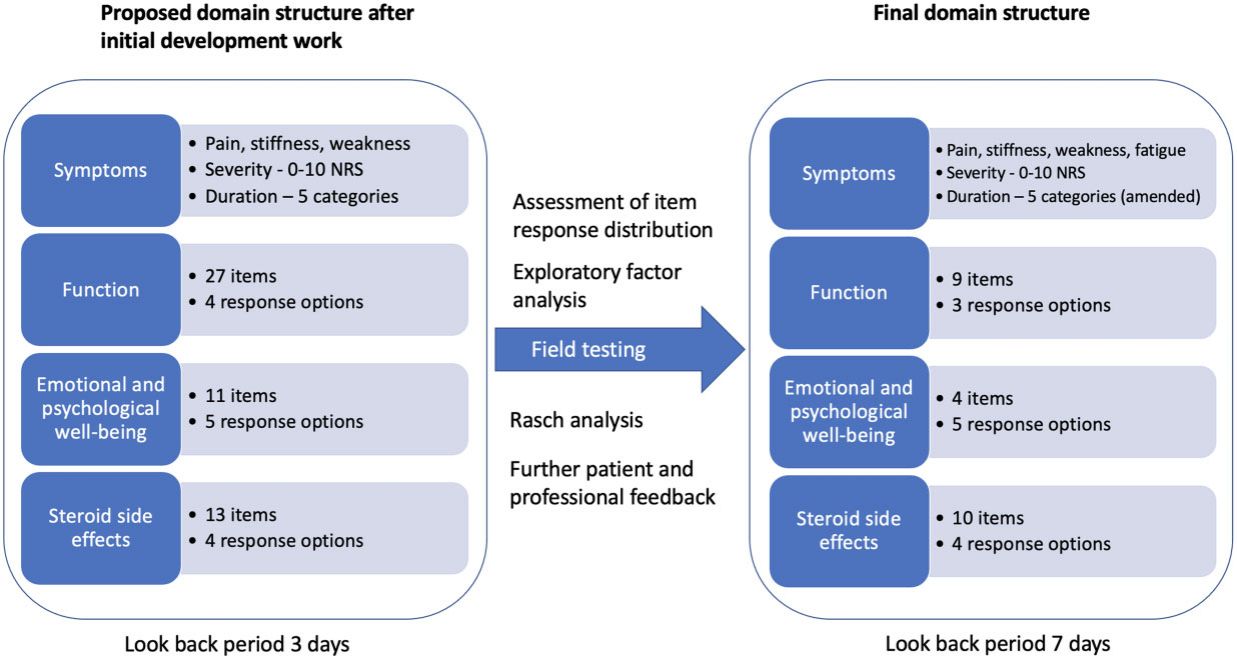
Development of the structure of the PMR-IS PMR-IS: PMR-Impact Scale; NRS: numerical rating scale.

Development of the conceptual framework, item development and pilot testing of the PMR-IS have been published elsewhere [6, 7]. Fig. 1 details the proposed structure of the PMR-IS after the initial development work. At this stage a long list of potential items was identified and a proposed domain structure covering symptoms, functional effects, psychological and emotional well-being, and steroid side effects was developed. Here we describe two studies that allowed further development and psychometric evaluation of the PROM.

### Patient and public involvement

The whole process of development of the PMR-IS was informed by consultation with people with PMR. Discussion with members of the PMRGCAuk North East support group (a regional patient support group affiliated to the charity PMRGCAuk) informed the initial idea for the PROM and this group contributed to the early development work. Trustees of the national PMRGCAuk charity helped refine the study design and participant materials for the field testing and evaluation studies. Two members of the study team (H.T. and S.M.) are members of the OMERACT PMR-SIG (a group working to develop a core outcome set for research studies of PMR) and have participated in regular discussions with patient partners throughout this process, which have increased understanding of patient perspectives and priorities.

### Field testing

Data for field testing were obtained via a cross-sectional postal survey. The North East–York Research Ethics Committee approved the study in April 2018 (REC reference 18/NE/0140).

#### Participant identification and sample size

Participating primary care practices from the West Midlands, UK carried out searches of their electronic patient databases to identify people with a coded diagnosis of PMR made within the preceding 2 years [8]. A clinician from the practice screened potential participants against inclusion/exclusion criteria, which included checking that the clinical features satisfied the core diagnostic criteria set out in the British Society for Rheumatology/British Society for Health Professionals in Rheumatology guidelines [9] and that the diagnosis had not subsequently been changed. People with GCA in addition to PMR were excluded. Details of full inclusion/exclusion criteria are given in Supplementary Data S1 (available at *Rheumatology* online). A sample of 250 respondents was aimed for to satisfy requirements for factor analysis (three to five times the number of respondents than number of items is recommended) [10] and Rasch analysis (where 250 is adequate for most purposes) [11].

Potential participants were sent a study pack containing a participant information leaflet and the questionnaires. No personally identifiable information was collected and return of the anonymized questionnaires was taken as implied consent to participate. To obtain responses representative of the entire disease course, participants were asked to complete the PMR-IS twice—once according to how they felt at the time of diagnosis and once according to how they felt now. This was a novel and pragmatic approach to mitigate the anticipated difficulties of recruiting sufficient numbers of incident cases through primary care. The two datasets, one for ‘at diagnosis’ data and one for ‘now’ data, were managed separately throughout. Analyses were conducted using SPSS [12] and the RUMM2020 Rasch analysis package [13].

#### Analysis

The distribution of item responses was examined to assess appropriateness of the labelling of response categories, frequencies of missing items, and risk of floor and ceiling effects.

The process of item reduction and determination of dimension structure for the functional and psychological domains was guided by exploratory factor analysis (EFA) and Rasch analysis [14].

EFA was conducted using principal component analysis with varimax rotation. The Kaiser–Meyer–Olkin [15] measure was used to verify the adequacy of the sample for analysis. Decisions on how many factors to retain were based on eigenvalues (retained if >1) and examining scree plots for point of inflection. Items with factor loading <0.5 onto a factor or loading >0.4 on more than one factor were excluded in an iterative process. When a unidimensional scale was created, Cronbach’s alpha was calculated as a measure of internal consistency. Further examination of item functioning, consideration of differential item functioning (DIF) and scale unidimensionality was undertaken using Rasch analysis.

Threshold plots were examined to ensure that response categories were ordered as expected. Unidimensionality was evaluated by identifying the two most different groups of items within the scale through principal component analysis of the residuals, thus producing the two most different estimates of person location for each individual. Independent t-tests were used to compare these person locations. The criterion for unidimensionality was that no >5% of the sample should have a significant (*P* < 0.05) difference in person location based on the two sets of items.

Overall fit was assessed by examining the item–trait interaction statistic, mean item and person fit residuals and the power of test-of-fit (based on the person-separation index). Individual item fit was assessed by studying item characteristic curves, chi-squared statistics for each item and item fit residuals. DIF by age, gender and duration since diagnosis was tested.

### Evaluation of measurement properties

A further cross-sectional postal survey was carried out to assess test–retest reliability, construct validity and responsiveness of the PMR-IS. The South Central–Hampshire B Research Ethics Committee approved the study in Oct 2019 (REC reference 19/SC/0525).

#### Participant identification and sample size

The same inclusion and exclusion criteria were used as for the field-testing study (S1), but participants were recruited from both primary and secondary care to increase the recruitment rate (primary care practices across the West Midlands and the rheumatology department of Midlands Partnership NHS Foundation Trust). Participants were asked to complete a baseline questionnaire booklet comprising the PMR-IS, the mHAQ [16] and the SF-36 [17]. Those that provided informed written consent to be contacted again were sent a second questionnaire booklet 2–6 weeks later, comprising a series of anchor questions and the PMR-IS. There were five anchor questions, one specific to each of the four domains and one on overall quality of life, and each had five response options (improved a lot, improved a little, stayed the same, worsened a little and worsened a lot).

A sample size of 200 was aimed for to achieve the recommended minimum of 50 participants remaining stable for the test–retest reliability analysis plus a large enough group whose condition changed between the two time points to allow responsiveness testing [18].

#### Analysis

Test–retest reliability for each domain was evaluated in the group reporting that they had ‘stayed the same’ on the anchor question for that specific domain. The intra-class correlation coefficient (ICC_agreement_), standard error of the measurement (SEM_agreement_) and the limits of agreement (LoA) were calculated for each domain.

Construct validity was assessed by testing pre-specified hypotheses about the strength and direction of correlation between scores on domains of the PMR-IS and scores on the comparator questionnaires. Responsiveness was evaluated by testing hypotheses about the expected mean change scores on domains of the PMR-IS in participants grouped according to their anchor question responses.

Consideration was also given to the interpretability of the measure. The risk of floor and ceiling effects was assessed by examining the frequencies of maximum and minimum responses and the smallest detectable change (SDC) at group level was calculated from the LoA.

## Results

### Field testing

#### Study sample and characteristics

A total of 256 participants returned paired questionnaires suitable for inclusion in the analysis. Demographic details are given in Table 1. Despite the search criteria for practices being to identify people diagnosed in the preceding 2 years, some respondents reported longer duration of diagnosis. We included the 14 participants who reported a date of diagnosis of between 2 and 5 years earlier but excluded any diagnosed >5 years earlier.

**Table 1:** Participant details

	Field testing study (*n* = 256)	Evaluation study (*n* = 210)
Mean (range) age (years)	73.9 (52.98)	72.2 (52.90)
Gender female [*n* (%)]	171 (67.1)	119 (57.1)
Mean (range) duration since diagnosis (months)	17.5 (1–60)	16.1 (1–36)
Percentage taking prednisolone	74.6	93.8
Mean (s.d.) dose of prednisolone (mg)	6.5 (5.1)	5.7 (4.3)

#### Distribution of item responses

Charts showing the distribution of responses to items in each domain are given in Supplementary Fig. S1 (available at *Rheumatology* online). Missing responses were <10% for all items.

In the symptoms and function domain, >10% of participants scored maximally on all the items ‘at diagnosis’ and minimally on all the items ‘now’, suggesting a risk of floor and ceiling effects. The responses in the ‘now’ data were more uniformly distributed.

Response categories for the symptom duration questions were amended as one option was used much less frequently than all the others. For the function domain, items for which missing or ‘not relevant’ responses were cumulatively >10% in either dataset were considered for removal. Seven items were excluded on this basis (all changes are detailed in Supplementary Fig. S1, available at *Rheumatology* online).

For the emotional and psychological domain, responses were more uniformly distributed and all response categories were used, therefore no items were removed at this stage.

For the steroid side effects domain, all response categories were used. Three items (high blood pressure, high blood sugar and cataracts) were removed as it was felt that these were not easily identifiable as directly related to prednisolone and may cause difficulties in reporting if they were pre-existing conditions.

#### Exploratory factor analysis

EFA of the ‘now’ function data, and the ‘now’ and ‘at diagnosis’ emotional and psychological well-being data found that these scales were unidimensional, and each had high internal consistency (Cronbach’s alpha >0.9). EFA of the ‘at diagnosis’ function data still resulted in two factors after iterative deletion of five items and there was no clinically meaningful distinction between the groups of items loading onto each factor. Therefore, Rasch analysis was used to aid further item reduction and more rigorous assessment of unidimensionality.

#### Rasch analysis

**Fig. 2:**
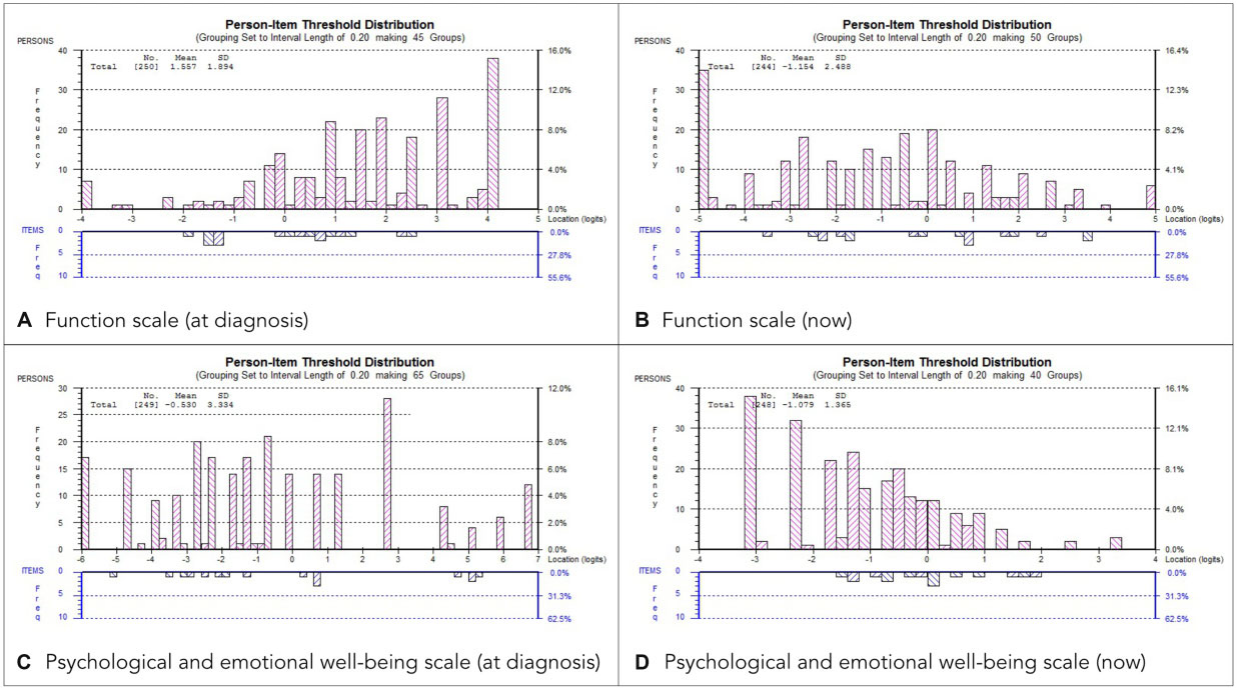
Person-item threshold distributions

A partial credit model [19] was used in each case and there were no disordered thresholds at any iteration. The least well-fitting items were iteratively deleted until unidimensional scales with satisfactory fit statistics were achieved. At the end of the process, a 9-item functional scale and a 4-item psychological and emotional well-being scale had been created. The only item showing DIF in the final scales was ‘take your shoes or socks on or off’, which showed DIF for gender in the ‘now’ dataset. Results of the Rasch analysis process are given in Supplementary Table S1 (available at *Rheumatology* online). Fig. 2 shows the person-item threshold distributions for the final scales.

### Final scale structure and scoring of the PMR-IS

Fig. 1 summarizes the developmental process and final scale structure of the PMR-IS. The full PMR-IS is available in Supplementary Data S2 (available at *Rheumatology* online). Fatigue was added to the symptoms domain after the field-testing study as on-going work with the OMERACT PMR-SIG [20] added to findings from previous research [6, 21] to support its status as a key symptom, rather than it being considered a component of psychological well-being. The ‘look-back period’ for the stem questions for each domain was initially set at 3 days but in response to patient and professional feedback, this was changed to 1 week prior to the evaluation study. The score for each domain is the mean item score converted to a percentage (higher scores indicate greater impact). As for the SF-36 [17], if fewer than half the items are completed for any domain, a score should not be calculated. Scores will be presented separately for each domain, rather than being combined to form an index, to aid clinical utility.

### Evaluation of measurement properties

#### Study sample and characteristics

A total of 210 first booklets and 179 paired booklets were eligible for inclusion in the analysis. Demographic details are given in Table 1. There were 25 respondents who reported being diagnosed >2 years ago. For this analysis we included the 11 participants who reported a diagnosis 2–3 years ago but excluded anyone diagnosed >3 years ago (*n* = 14). This was felt to strike the optimal balance of maximizing participant numbers whilst keeping the study population representative of ‘typical’ PMR.

#### Test–retest reliability

A sample size of >50 was achieved for each domain. The ICC_agreement_ was >0.8 in each domain, suggesting good reliability [22]. The SEM_agreement_ for each domain ranged from 9.3 to 11.9 on a scale out of 100 (see Table 2).

**Table 2:** Test–retest reliability and smallest detectable change results

Scale	*n*	ICC_agreement_ (95% CI)	SEM_agreement_	Mean and LoA	SDC	
					Individual	Group
Symptoms	59	0.83 (0.73, 0.90)	11.85	4.22 (−27.88, 36.32)	32.10	4.18
Function	80	0.85 (0.77, 0.90)	8.44	0.67 (−22.83, 24.16)	23.50	2.63
Emotional and psychological well-being	95	0.81 (0.73, 0.87)	9.72	1.05 (−25.97, 28.07)	27.02	2.77
Steroid side effects	100	0.83 (0.76, 0.88)	9.31	−1.94 (−27.59, 23.72)	25.66	2.57

*n*: number of participants reporting they had ‘stayed the same’ on this scale between completing the two questionnaires; SEM_agreement_: standard error of the measurement, calculated for agreement; ICC_agreement_: intraclass correlation coefficient, two-way, calculated for agreement; LoA: limits of agreement between which 95% of second values are expected to fall calculated using the Bland and Altman method [23]; SDC: smallest detectable change. SDC_ind_ = 1.96 × √2 × SEM. SDC_group_ = SDC_ind_/√n.

#### Construct validity

Ten out of 11 hypotheses were satisfied (Supplementary Table S2, available at *Rheumatology* online). The PMR-IS therefore met the criteria of >75% of hypotheses being satisfied to demonstrate good construct validity [22].

#### Responsiveness

**Fig. 3:**
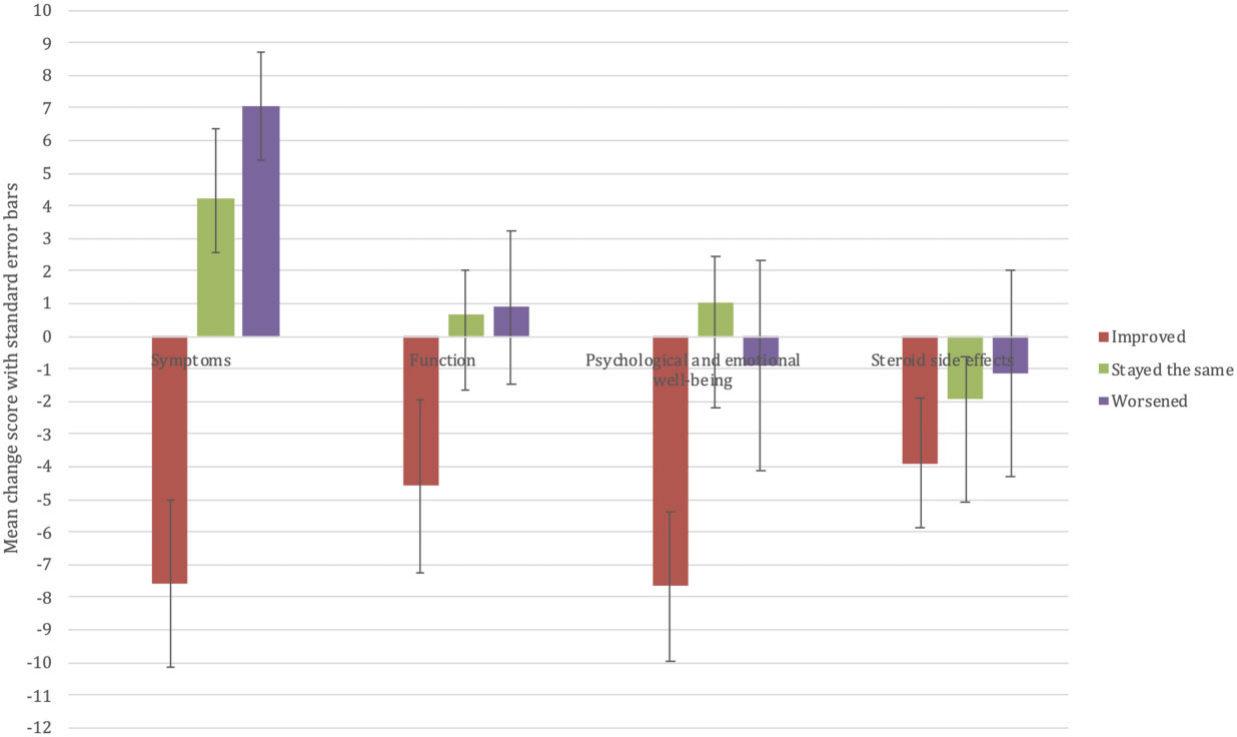
Bar chart of mean change scores per domain for groups defined by participants’ response to the domain-specific anchor question

Due to the small numbers of participants in each anchor question response group, the ‘worsened/improved a little’ and ‘worsened/improved a lot’ groups were combined into ‘worsened’ and ‘improved’ categories for each domain. Four out of five hypotheses about the expected trends in change scores were satisfied and the PMR-IS scores for each domain changed as expected for the group that rated themselves ‘improved’. However, for the ‘worsened’ group, the mean change scores were small with high variability (see Fig. 3). Supplementary Table S3 (available at *Rheumatology* online) contains full results for responsiveness testing.

#### Interpretability

In the function and psychological and emotional well-being domains there was a floor effect, with >15% participants scoring at the minimum. The SDC at group level for each domain is given in Table 2.

## Discussion

We have developed a new PROM, the PMR-IS, which has good construct validity and test–retest reliability in people with PMR. The outcome measure was derived from qualitative data exploring the patient experience and has been tested and refined at each stage based on responses from people with the condition.

PROMs are increasingly recognized as valid and responsive tools by which to measure outcomes in a wide variety of conditions [24, 25]. In clinical trials, the use of PROMs in addition to traditional clinical indicators allows the patient perspective of the physical, functional and psychological impact of a disease to be systematically captured and therefore the impact of the intervention to be more comprehensively assessed. PROMs can also allow the patient perspective to be incorporated into other study types—routine collection of PROMs into the electronic health record could enable inclusion of this information into big data longitudinal and cross-sectional observational research [26, 27]. In clinical practice, PROMs can be used at an individual level in guiding patient assessment and management, informing treatment decisions and follow-up schedules, and facilitating supported self-management [28, 29].

PMR lends itself to patient-reported assessment because of the nature of its symptoms and effects and the balance that has to be struck between the effects of the disease and the adverse effects of treatment. Until now, there has been no valid, disease-specific outcome measure for the condition that incorporates patient experiences, despite repeated assertions that this is an unmet need [21, 30–32].

The process of developing and refining the scale structure of the PMR-IS was stepwise and rigorous, and built on a strong theoretical understanding of the conceptual framework derived from qualitative exploration of patient experiences of the condition. One of the challenges in ‘measuring’ outcomes in PMR is the need to capture the severity of symptoms at onset and fluctuations around a much lower level of symptoms over the duration of the disease course. To ensure that the PMR-IS contained items applicable to people in the early stages of the disease, we asked people to retrospectively complete the score, thinking back to how they felt at onset. This carries a risk of recall bias and bias due to response shift [33] but was a pragmatic approach given the anticipated difficulties of recruiting people newly diagnosed with PMR. Further evaluation of responsiveness of the PMR-IS, for example validation in a longitudinal cohort, is needed to confirm that this approach led to inclusion of a sufficient range of items that work across the disease course.

Refinement of the function and psychological and emotional well-being scores involved application of both classical and modern test theory methods. The benefits of using Rasch in this study were verifying ordering of response categories, providing a more powerful study of item functioning, rigorous assessment of unidimensionality and enabling testing for DIF.

Once an instrument has been developed it needs to be evaluated in the population in which it will be used. This is not a one-off assessment, it is a process of gathering evidence to support or refute the reliability, validity and responsiveness of the instrument in defined circumstances. The evaluation study presented here is the first step in the process of gathering evidence to support the use of the PMR-IS. Good construct validity and test–retest reliability have been demonstrated. This initial study also provides some evidence that the PMR-IS is a responsive measure for detecting improvement in PMR but the numbers of participants in the responsiveness analysis were too small to be confident in the ability of the tool to detect worsening in the condition.

In addition to an instrument’s psychometric properties, consideration needs to be given to the interpretability of the scores in the population of interest. Our results show a risk of floor effects in the function and psychological and emotional well-being domains of the PMR-IS. However, this same limitation has been found for pain and stiffness VAS, the HAQ and the mHAQ in PMR, and is to be expected given the clinical course of the condition [28]. This might not cause significant difficulty in a clinical trial as once the participant is scoring within the ‘floor effect’ margins, the condition might reasonably be considered to be under control and further differentiation may not be needed. If discrimination of people with low levels of these constructs was required, further items would have to be developed and added but this would have to be balanced against increased burden for participants. In future, the use of item banks and computer adaptive testing may allow targeted questions but the technology to do this is not currently available.

Two key parameters for interpretability of an instrument or scale are the SDC and the minimally important change. The SDC value is derived from the LoA from the reliability analysis. At an individual level the results here are high but at group level they are reasonable, at between 2–4% for each domain. Further studies are needed to evaluate the minimally important change for patients and to ensure that the scales are sufficiently sensitive to detect this.

The PMR-IS is the first composite PROM for PMR. It has the potential to facilitate better research into PMR by ensuring that researchers measure outcomes that truly matter to patients. In future we envisage that it could also be used in clinical practice to aid shared decision making and empower people to be more involved in management of their condition. It has good construct validity and test–retest reliability in the target population and can detect improvement in the condition. Further evaluation of the PMR-IS in longitudinal cohort studies and clinical trials will allow assessment of its performance in detecting relapse and remission, and provide more precise estimates of its interpretability parameters.

## Supplementary Material

keac317_Supplementary_Data

## Data Availability

Individual participant data that underlie the results reported in this article, after de-identification (text, tables, figures and appendices) will be made available to researchers who provide a methodologically sound proposal, to achieve the aims in the approved proposal. Requests for access to the data should be made to the corresponding author.

## References

[1] González-Gay MA , Matteson EL, Castañeda S. Polymyalgia rheumatica. Lancet 2017;390:1700–12. 28774422 10.1016/S0140-6736(17)31825-1

[2] Crowson CS , Matteson E, Myasoedova E et al The lifetime risk of adult-onset rheumatoid arthritis and other inflammatory autoimmune rheumatic diseases. Arthritis Rheum 2011;63:633–9. 21360492 10.1002/art.30155 PMC3078757

[3] Partington RJ , Muller S, Helliwell T, Mallen CD, Abdul Sultan A. Incidence, prevalence and treatment burden of polymyalgia rheumatica in the UK over two decades: A population-based study. Ann Rheum Dis 2018;77:1750–6. 30297332 10.1136/annrheumdis-2018-213883

[4] Dejaco C , Singh YP, Perel P et al; American College of Rheumatology. 2015 recommendations for the management of polymyalgia rheumatica: A European League Against Rheumatism/American College of Rheumatology collaborative initiative. Ann Rheum Dis 2015;74:1799–807. 26359488 10.1136/annrheumdis-2015-207492

[5] Twohig H , Owen C, Muller S et al Outcomes measured in polymyalgia rheumatica and measurement properties of instruments considered for the OMERACT Core Outcome Set: A Systematic Review. J Rheumatol 2021;48:883–93. 32739892 10.3899/jrheum.200248

[6] Twohig H , Mitchell C, Mallen C, Adebajo A, Mathers N. “I suddenly felt I’d aged”: A qualitative study of patient experiences of polymyalgia rheumatica (PMR). Patient Educ Couns 2015;98:645–50. 25638304 10.1016/j.pec.2014.12.013

[7] Twohig H , Jones G, Mackie S, Mallen C, Mitchell C. Assessment of the face validity, feasibility and utility of a patient-completed questionnaire for polymyalgia rheumatica: A postal survey using the QQ-10 questionnaire. Pilot Feasibil Stud 2018;4:7. 10.1186/s40814-017-0150-y PMC5501557 28694986

[8] NICE. Polymyalgia rheumatica | Health topics A to Z | CKS | NICE 2021 https://cks.nice.org.uk/topics/polymyalgia-rheumatica/ (September 2021, date last accessed).

[9] Dasgupta B , Borg F, Hassan N et al; BSR and BHPR Standards, Guidelines and Audit Working Group. BSR and BHPR guidelines for the management of polymyalgia rheumatica. Rheumatology (Oxford) 2010;49:186–90. 19910443 10.1093/rheumatology/kep303a

[10] Norman G , Streiner D. Biostatistics: The bare essentials. Hamilton; Lewiston, NY: B.C. Decker, 2008.

[11] Linacre JM. Sample size and item calibration or person measure stability. Rasch Measur Trans 1994; 7: 328.

[12] IBM Corp. Released 2020. IBM SPSS Statistics for Macintosh, Version 27.0. Armonk, NY: IBM Corp, 2020.

[13] Andrich D , Sheridan B, Luo G. RUMM2020: Rasch Unidimensional Measurement Models. [Computer software] 2003.

[14] Rasch G. Probabilistic models for some intelligence and achievement tests. Chicago: MESA Press, 1960.

[15] Kaiser HF. A second generation little jiffy. Psychometrika 1970;35:401–15.

[16] Maska L , Anderson J, Michaud K. Measures of functional status and quality of life in rheumatoid arthritis: Health Assessment Questionnaire Disability Index (HAQ), Modified Health Assessment Questionnaire (MHAQ), Multidimensional Health Assessment Questionnaire (MDHAQ), Health Assessment. Arthrit Care Res 2011;63:4–13. 10.1002/acr.20620 22588760

[17] Ware JEJ , Sherbourne CD. The MOS 36-item short-form health survey (SF-36). I. Conceptual framework and item selection. Med Care 1992;30:473–83. 1593914

[18] Mokkink LB , de Vet HB, Prinsen C et al COSMIN risk of bias checklist for systematic reviews of patient-reported outcome measures. Qual Life Res 2018;27:1171–9. 29260445 10.1007/s11136-017-1765-4 PMC5891552

[19] Masters GN. A RASCH model for partial credit scoring. Psychometrika 1982;47:149–74.

[20] Yates M , Owen C, Muller SM et al; OMERACT PMR Working Group. Feasibility and face validity of outcome measures for use in future studies of polymyalgia rheumatica: An OMERACT study. J Rheumatol 2020;47:1379–84. 32007937 10.3899/jrheum.190575

[21] Matteson EL , Maradit-Kremers H, Cimmino M et al Patient-reported outcomes in polymyalgia rheumatica. J Rheumatol 2012;39:795–803. 22422492 10.3899/jrheum.110977

[22] Terwee CB , Bot S, de Boer MR et al Quality criteria were proposed for measurement properties of health status questionnaires. J Clin Epidemiol 2007;60:34–42. 17161752 10.1016/j.jclinepi.2006.03.012

[23] Bland JM , Altman DG. Statistical methods for assessing agreement between two methods of clinical measurement. Lancet 1986;1:307–10. 2868172

[24] Devlin NJ , Appleby J, Buxton M, Vallance-Owen A. Getting the most out of PROMS. Putting health outcomes at the heart of NHS decision making. London: The King's Fund, 2010; www.kingsfund.org.uk/publications.

[25] Mercieca-Bebber R , King MT, Calvert MJ, Stockler MR, Friedlander M. The importance of patient-reported outcomes in clinical trials and strategies for future optimization. Patient Relat Outcome Meas 2018;9:353–67. 30464666 10.2147/PROM.S156279 PMC6219423

[26] Calvert M , Thwaites R, Kyte D, Devlin N. Putting patient-reported outcomes on the ‘Big Data Road Map.’ J R Soc Med 2015;108:299–303. 25827908 10.1177/0141076815579896 PMC4535436

[27] Calvert M , Kyte D, Price G, Valderas JM, Hjollund NH. Maximising the impact of patient reported outcome assessment for patients and society. BMJ 2019;364:k5267. 30679170 10.1136/bmj.k5267

[28] Greenhalgh J. The applications of PROs in clinical practice: What are they, do they work, and why? Qual Life Res 2009;18:115–23. 19105048 10.1007/s11136-008-9430-6

[29] Boyce MB , Browne JP, Greenhalgh J. The experiences of professionals with using information from patient-reported outcome measures to improve the quality of healthcare: A systematic review of qualitative research. BMJ Qual Saf 2014;23:508–18. 10.1136/bmjqs-2013-002524 24505110

[30] Emamifar A , Hess S, Ellingson T et al Clinical presentation and treatment response in patients with polymyalgia rheumatica and giant cell arteritis during a 40-week follow-up. Rheumatol Adv Pract 2011;5:1–17. 10.1093/rap/rkab091 PMC8665449 34909566

[31] Owen CE , Yates M, Twohig H et al Toward a core outcome measurement set for polymyalgia rheumatica: Report from the OMERACT 2018 special interest group. J Rheumatol 2019;46:1360–4. 30709960 10.3899/jrheum.181050

[32] Camellino D , Matteson EL, Buttgereit F, Dejaco C. Monitoring and long-term management of giant cell arteritis and polymyalgia rheumatica. Nat Rev Rheumatol 2020;16:481–95. 32759996 10.1038/s41584-020-0458-5

[33] Sprangers MA. Response-shift bias: A challenge to the assessment of patients’ quality of life in cancer clinical trials. Cancer Treatment Rev 1996;22:55–62. 10.1016/s0305-7372(96)90064-x 8625350

