# Supplementary material for: Development and psychometric evaluation of the PMR-Impact Scale: a new patient reported outcome measure for polymyalgia rheumatica
Source: Rheumatology (Oxford). 2022 May 26;62(2):758–65. doi: 10.1093/rheumatology/keac317 (PMC9891434; doi:10.1093/rheumatology/keac317)
Supplement: keac317_Supplementary_Data [file keac317_supplementary_data.docx]

**Supplementary material for the paper:** **Development and psychometric evaluation of the PMR-Impact Scale: a new patient reported outcome measure for polymyalgia rheumatica**

**Contents**

| Supplementary Data S1- Inclusion and Exclusion criteria / recruitment process | Page 2 |
| --- | --- |
| Supplementary Figure S1 – Distribution of item responses from the field-testing study | Page 3 |
| Supplementary Table S1 – Rasch results tables | Page 9 |
| Supplementary Data S2 – The PMR-IS | Page 11 |
| Supplementary Table S2 – Hypothesis testing for construct validity | Page 18 |
| Supplementary Table S3 – Results for responsiveness analysis | Page 20 |

**Supplementary Data S1 – Inclusion and Exclusion criteria / recruitment process**

Practices were asked to run a search of their patient databases to identify people diagnosed with PMR within the preceding two years. Either a GP or nurse from the practice or a member of the Clinical Research Network team with appropriate access rights, then screened this list of patients against the inclusion and exclusion criteria.

**Inclusion criteria:**

To be included, people needed a diagnosis of PMR made within the previous 2 years and not subsequently changed.

The diagnosis should be supported by the following features, which are based on the British Society for Rheumatology / British Society for Health Professionals in Rheumatology guidelines:^9^

- - Age > 50 years.
  - Bilateral shoulder or pelvic girdle aching or both for at least 2 weeks.
  - Morning stiffness.
  - Evidence of an acute phase response (raised ESR / CRP).
  - Diagnosis made by a rheumatologist despite the presence of atypical features (e.g. normal ESR / CRP).

**Exclusion criteria:**

- Diagnosis of Giant Cell Arteritis
- Inability to read / write English well enough to understand the instructions and complete the questionnaire.
- Comorbidities that made an invitation to participate in the study inappropriate in the view of the participant’s GP (dementia, significant anxiety / depression, receiving end of life care etc.).

**Supplementary Figure S1 – Distribution of item responses from the field-testing study**

**Bar charts showing distribution of responses to symptoms items**

| 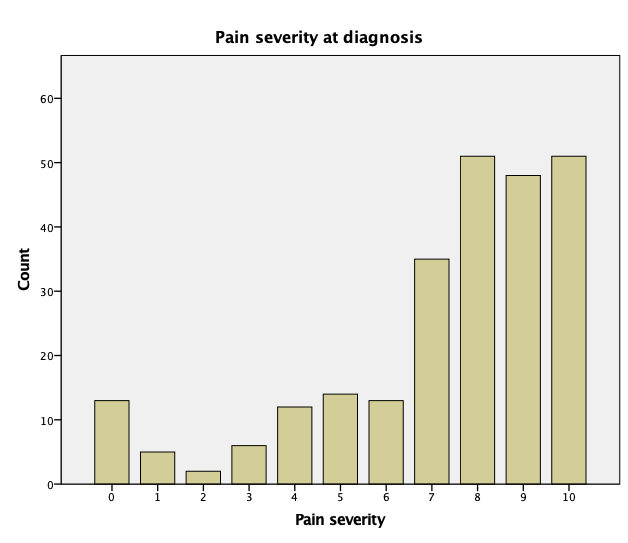 | 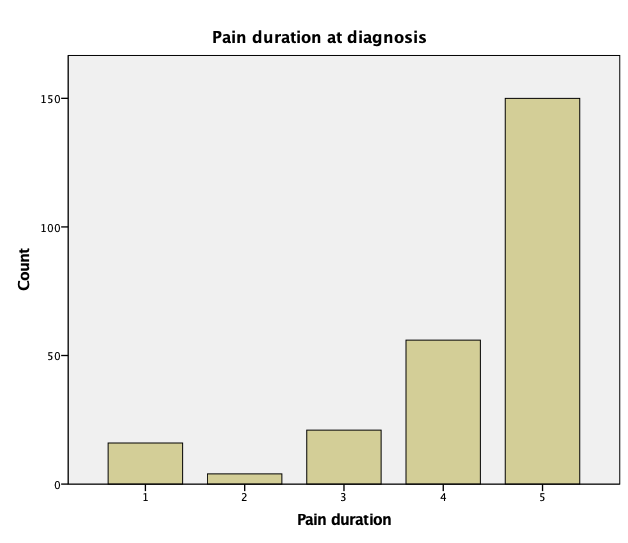 |
| --- | --- |
| 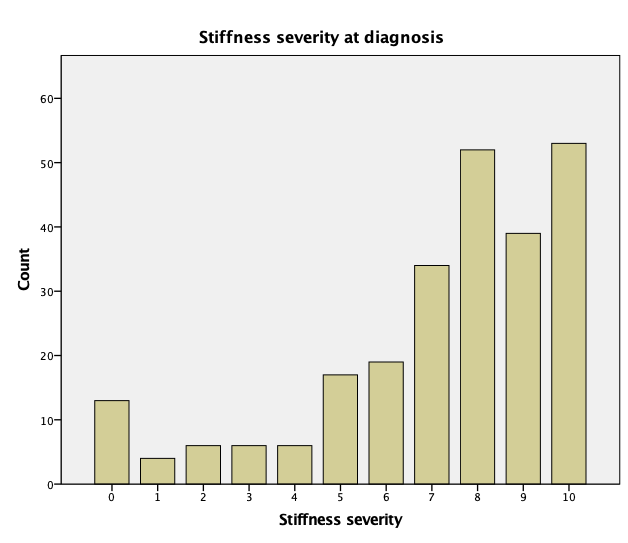 | 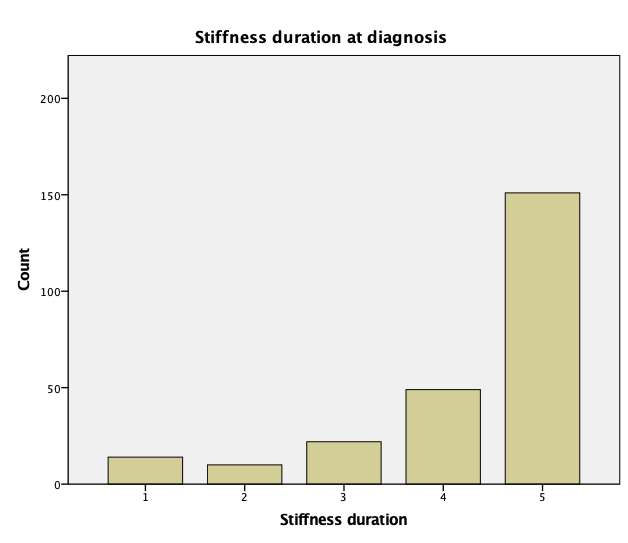 |
| 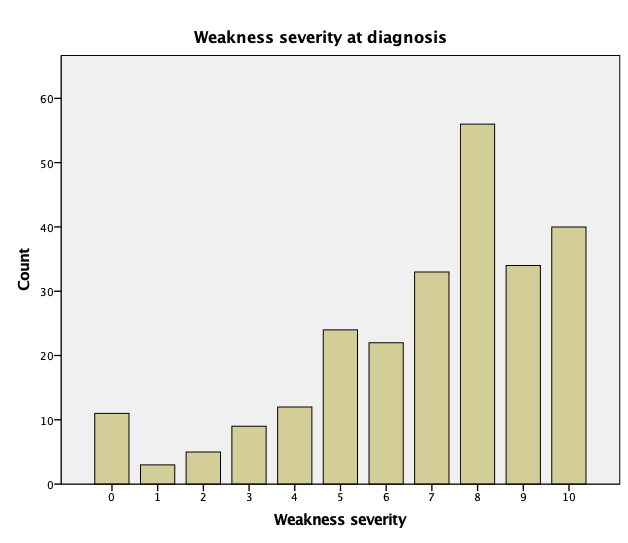 | 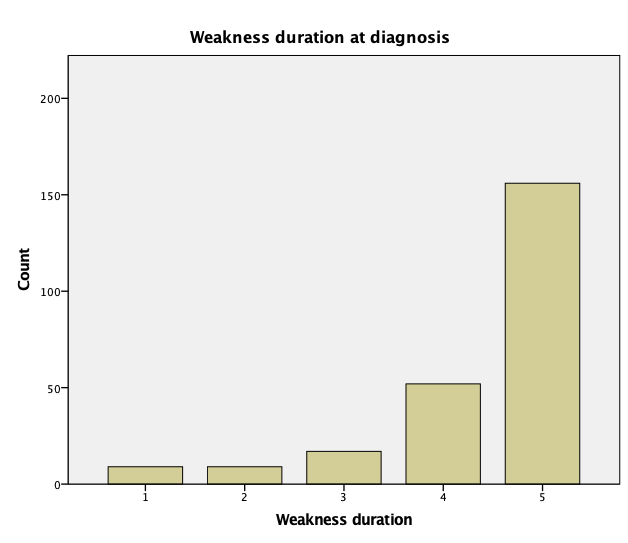 |

| 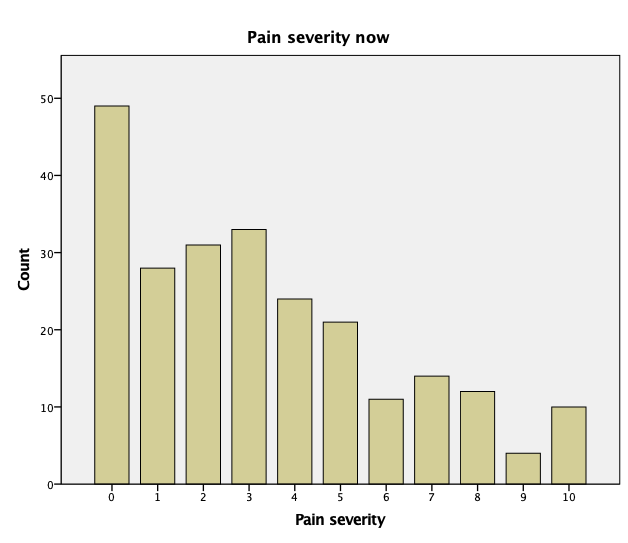 | 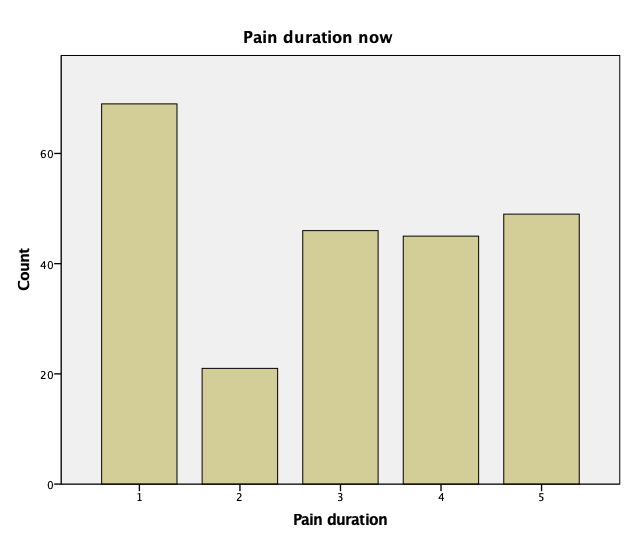 |
| --- | --- |
| 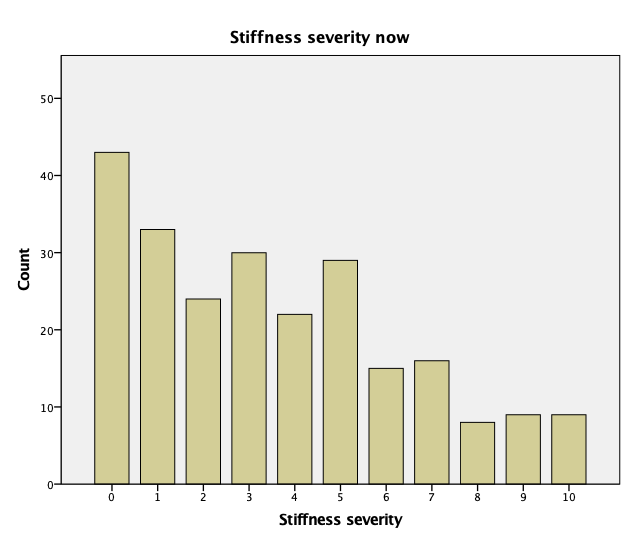 | 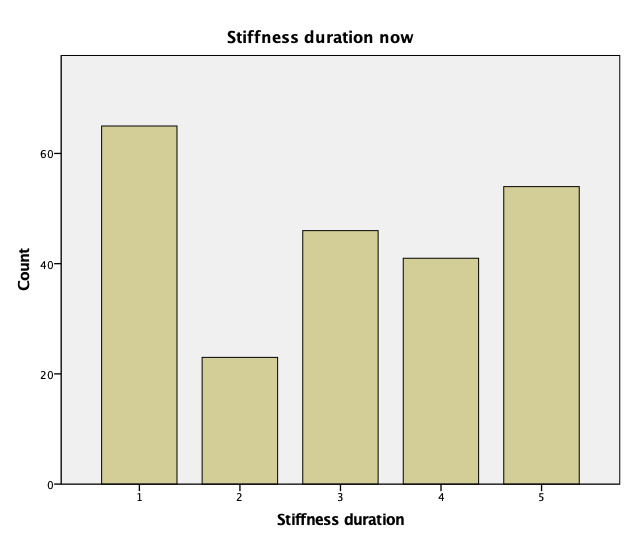 |
| 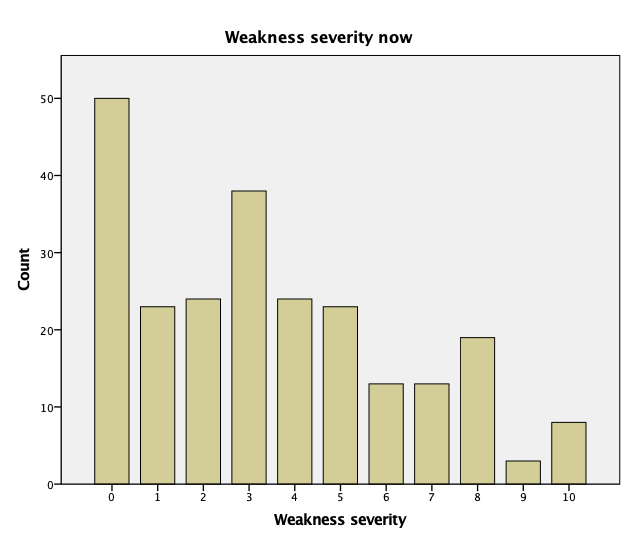 | 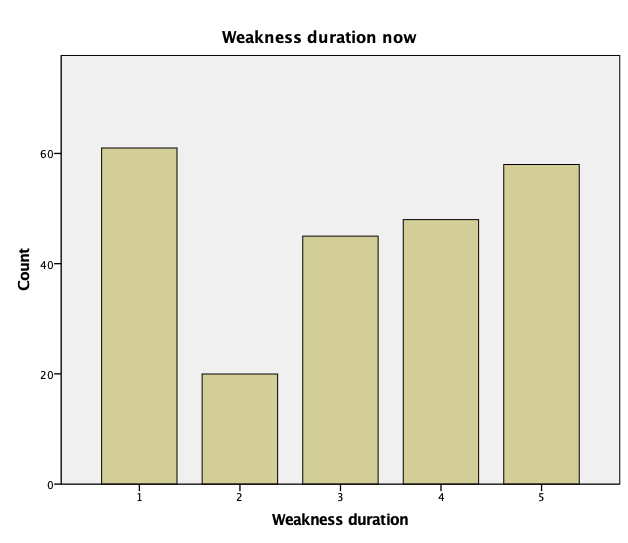 |

Question asked on severity: how severe has the (pain / stiffness / weakness) from your PMR been during the last 3 days?

Responses options: visual analogue scale (VAS), scored from 0-10 where 0 = no pain and 10 = the worst pain you have ever felt.

Question asked on duration**:** on average much of each day has the (pain / stiffness / weakness) from your PMR been present for during the last 3 days?

Response options: 1 = less than 30 mins, 2 = less than 1 hour, 3 = around 1-3 hours, 4 = about half the day, 5 = all day

Based on these results, response options were altered such that

0 = none, 1 = less than 1 hour, 2 = around 1-3 hours, 4 = all day

**Bar charts depicting distribution of responses to functional activity items**

Question asked**:** Over the last 3 days, compared to what you can normally do, has PMR limited your ability to do the following activities?

Items removed due to having >10% missing or not relevant responses

- In / out bath
- Intimate activities
- Gardening
- Sports / hobbies
- Walk up hills
- Housework
- Driving

NB. Walking upstairs was left in as it was felt to be such an important functional activity where it was relevant.

**Bar charts depicting distribution of responses to emotional and psychological well-being items**

Question asked**:** Over the last 3 days, have your PMR symptoms….?

**Bar charts depicting distribution of responses to steroid side effects items**

Question asked**:** Over the last 3 days, have you had any of the following side effects from your prednisolone medication?

**Supplementary Table S1 – Rasch results tables**

**Summary of results for (at diagnosis) functional scale fit to a Rasch model**

| **Scale iteration** | **Number of items** | **Targeting** | **Unidimensionality** | | **Item fit residuals** | | **Person fit residuals** | | **Item trait interaction statistic** | **Power of test of fit** |
| --- | --- | --- | --- | --- | --- | --- | --- | --- | --- | --- |
|  |  |  | **% significant t-tests** | **Unidimensional?** | **Mean** | **SD** | **Mean** | **SD** |  |  |
| 1 | 20 | 1.31 | 13.6 | No | -0.26 | 1.52 | -0.28 | 1.35 | Significant | Excellent |
| 2 | 18 | 1.45 | 11.3 | No | -0.24 | 1.38 | -0.33 | 1.41 | Significant | Excellent |
| 3 | 15 | 1.45 | 9.6 | No | -0.18 | 1.19 | -0.28 | 1.26 | Not significant | Excellent |
| 4 | 13 | 1.40 | 5.8 | No | -0.15 | 1.37 | -0.27 | 1.17 | Not significant | Excellent |
| 5 | 11 | 1.41 | 4.0 | Yes | -0.12 | 1.24 | -0.29 | 1.13 | Not significant | Excellent |
| 6 | 9 | 1.56 | 2.6 | Yes | -0.14 | 0.68 | -0.31 | 1.12 | Not significant | Excellent |

**Summary of results for (now) functional scale fit to a Rasch model**

| **Scale iteration** | **Number of items** | **Targeting** | **Unidimensionality** | | **Item fit residuals** | | **Person fit residuals** | | **Item trait interaction statistic** | **Power of test of fit** |
| --- | --- | --- | --- | --- | --- | --- | --- | --- | --- | --- |
|  |  |  | **% significant t-tests** | **Unidimensional?** | **Mean** | **SD** | **Mean** | **SD** |  |  |
| 1 | 20 | -1.44 | 12.0 | No | -0.37 | 1.76 | -0.36 | 1.37 | Significant | Excellent |
| 2 | 11 | -1.56 | 5.5 | No | -0.50 | 0.93 | -041 | 1.14 | Significant | Excellent |
| 3 | 9 | -1.15 | 3.5 | Yes | -0.43 | 1.06 | -0.38 | 1.07 | Significant (0.04) | Excellent |

NB. The highlighted rows show the scale iterations where a good fit was achieved

**Final 9 items:** Get in or out of a car, get in or out of bed, turn over in bed, wash yourself fully, put on or take off your shoes and socks, get on or off the toilet, walk up or down stairs, carry or lift thigs, reach up above your head

**Summary of results for (at diagnosis) emotional and psychological well-being scale fit to a Rasch model**

| Scale iteration | Number of items | Targeting | Unidimensionality | | Item fit residuals | | Person fit residuals | | Item trait interaction statistic | Power of test of fit |
| --- | --- | --- | --- | --- | --- | --- | --- | --- | --- | --- |
|  |  |  | % significant t-tests | Unidimensional? | Mean | SD | Mean | SD |  |  |
| 1 | 11 | -0.24 | 9.7 | No | -0.50 | 2.72 | -0.59 | 1.67 | Significant | Excellent |
| 2 | 8 | -0.4 | 10.7 | No | -0.42 | 2.49 | -0.56 | 1.48 | Significant | Excellent |
| 3 | 7 | -0.54 | 12.6 | No | -0.11 | 1.93 | -0.6 | 1.51 | Not significant | Excellent |
| 4 | 4 | -0.53 | 3.2 | Yes | -0.59 | 1.11 | -0.81 | 1.45 | Not significant | Excellent |

**Summary of results for (now) emotional and psychological well-being scale fit to a Rasch model**

| Scale iteration | Number of items | Targeting | Unidimensionality | | Item fit residuals | | Person fit residuals | | Item trait interaction statistic | Power of test of fit |
| --- | --- | --- | --- | --- | --- | --- | --- | --- | --- | --- |
|  |  |  | % significant t-tests | Unidimensional? | Mean | SD | Mean | SD |  |  |
| 1 | 11 | -1.77 | 9.4 | No | -0.12 | 1.78 | -0.30 | 1.18 | Significant | Excellent |
| 2 | 4 | -3.53 | 2.3 | Yes | -0.35 | 1.17 | -0.60 | 1.20 | Not significant | Excellent |
| 3 | 5 | -1.44 | 3.0 | Yes | -0.21 | 1.27 | -0.30 | 0.91 | Significant | Excellent |
| 4 | 4 | -1.08 | 0.0 | Yes | -0.08 | 0.97 | -0.42 | 1.16 | Not significant | Good |

**Final 4 items:** caused you to feel low in mood, caused you to feel anxious, caused you to feel vulnerable, lowered your self confidence

**Supplementary Data S2: The PMR-IS**


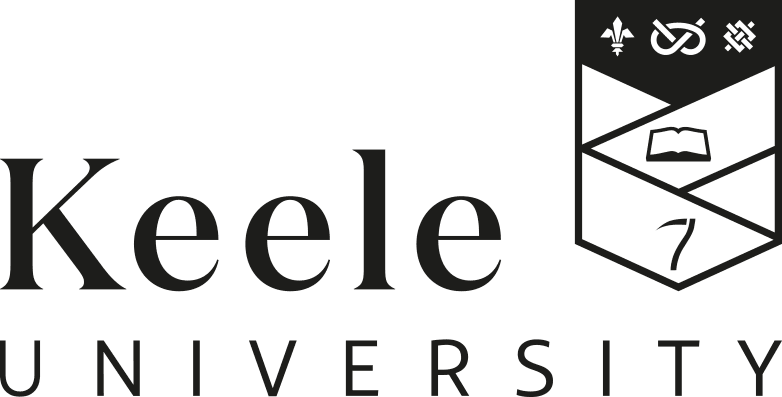


**The Polymyalgia Rheumatica Impact Scale (PMR-IS)**

The following questionnaire asks about your symptoms of polymyalgia rheumatica (PMR) and the way in which it is affecting you at the moment.

If you are unsure about how to answer a question, please give the best answer you can.

Thank you.

**Today’s date:**

| **PERSONAL DETAILS** | | |
| --- | --- | --- |
| **Name** |  | |
|  |  |  |
| **Date of birth** |  |  |
|  |  |  |
| **Gender** | **Male** | **□** |
|  | **Female** | **□** |
|  |  |  |
| **Length of time since PMR diagnosis** |  |  |
| **Have you been referred to a rheumatologist about your** | **Yes** | **□** |
| **PMR?** | **No** | **□** |
| **Are you taking prednisolone?** | **Yes □**  **No □** | |
| **Current dose of prednisolone if taking:** |  | **mg** |
| **Other current medication** |  |  |
|  |  |  |
|  |  |  |
|  |  |  |
| **Other therapy you are having for your PMR e.g. physiotherapy:** |  |  |

1. **Symptoms**

Thinking about how your **PMR** has affected you **during the last week**, please answer the following questions by circling one response

**Pain**

How bad has the pain **caused by your PMR** been during the last week?

| **0** | **1** | **2** | **3** | **4** | **5** | **6** | **7** | **8** | **9** | **10** |
| --- | --- | --- | --- | --- | --- | --- | --- | --- | --- | --- |
| **No pain** | | | | | **Severe pain** | | | | | |

On average, how much of each day has the pain been present for?

| ***None*** | ***Less than 1 hour*** | ***Around 1-3 hours*** | ***About half the day*** | ***All day*** |
| --- | --- | --- | --- | --- |

**Stiffness**

How bad has the stiffness **caused by your PMR** been during the last week?

| **0** | **1** | **2** | **3** | **4** | **5** | **6** | **7** | **8** | **9** | **10** |
| --- | --- | --- | --- | --- | --- | --- | --- | --- | --- | --- |
| **No stiffness** | | | | | **Severe stiffness** | | | | | |

On average, how much of each day has the stiffness been present for?

| ***None*** | ***Less than 1 hour*** | ***Around 1-3 hours*** | ***About half the day*** | ***All day*** |
| --- | --- | --- | --- | --- |

**Weakness**

How much weakness **caused by your PMR** have you experienced during the last week?

| **0** | **1** | **2** | **3** | **4** | **5** | **6** | **7** | **8** | **9** | **10** |
| --- | --- | --- | --- | --- | --- | --- | --- | --- | --- | --- |
| **No weakness** | | | | | **Severe weakness** | | | | | |

On average, how much of each day has any weakness been present for?

| ***None*** | ***Less than 1 hour*** | ***Around 1-3 hours*** | ***About half the day*** | ***All day*** |
| --- | --- | --- | --- | --- |

**Fatigue**

How bad has the fatigue **caused by your PMR** been in the last week?

| **0** | **1** | **2** | **3** | **4** | **5** | **6** | **7** | **8** | **9** | **10** |
| --- | --- | --- | --- | --- | --- | --- | --- | --- | --- | --- |
| **No fatigue** | | | | | **Severe fatigue** | | | | | |

On average, how much of each day have you felt fatigued?

| ***None*** | ***Less than 1 hour*** | ***Around 1-3 hours*** | ***About half the day*** | ***All day*** |
| --- | --- | --- | --- | --- |

**2*.*** **Function**

Over the last week, has **your PMR** limited your ability to do the following activities?

Please put a cross in one box on each line

|  | **Not limited at all** | **Moderately Limited** | **Severely Limited** |
| --- | --- | --- | --- |
| Get in or out of a car |  |  |  |
| Get in or out of bed |  |  |  |
| Turnover in bed |  |  |  |
| Wash yourself fully |  |  |  |
| Put on or take off your socks and shoes |  |  |  |
| Get on or off the toilet |  |  |  |
| Walk up or down stairs |  |  |  |
| Carry or lift things |  |  |  |
| Reach above your head for things |  |  |  |

**3. Emotional and psychological well-being**

In the last week have **your PMR** symptoms caused any of the following feelings?

Please put a cross in one box on each line

|  | **No, not at all** | **A little of the time** | **Some of the time** | **Most of the time** | **All of the time** |
| --- | --- | --- | --- | --- | --- |
| Caused you to feel low in mood? |  |  |  |  |  |
| Caused you to feel anxious? |  |  |  |  |  |
| Caused you to feel vulnerable? |  |  |  |  |  |
| Lowered your self-confidence? |  |  |  |  |  |

**4. Treatment side effects**

In the last week, have you had any of the following side effects from **your prednisolone** medication?

Please put a cross in one box on each line.

|  | **No, I’m not affected by this** | **Yes, but I’m not bothered by it** | **Yes, and it’s affected me a little** | **Yes, and it’s affected me a lot** |
| --- | --- | --- | --- | --- |
| Weight gain |  |  |  |  |
| Change in appearance (fatter face, saggy skin) |  |  |  |  |
| Sleep disturbance |  |  |  |  |
| Stomach upset or heartburn |  |  |  |  |
| Mood disturbance |  |  |  |  |
| Increased appetite |  |  |  |  |
| Muscle weakness |  |  |  |  |
| Easily bruised or thin skin |  |  |  |  |
| Swelling of the feet or ankles |  |  |  |  |
| Hair loss |  |  |  |  |

**Thank you very much for completing this questionnaire**

**Supplementary Table S2 – Hypothesis testing for construct validity**

| **Comparator construct** | **Hypotheses** | **Results**  **(Spearman correlation)** | **Interpretation** |
| --- | --- | --- | --- |
| Symptom severity | The symptoms score of the PMR-IS is moderately to highly negatively correlated (-0.5 to -0.7) with the bodily pain and energy / fatigue scores of the SF-36. | -0.81 with SF-36 bodily pain (n=206)  -0.66 with SF-36 energy / fatigue (n=204) | 2 of 2 hypotheses met |
| Physical function | The score from the functional domain on the PMR-IS is strongly positively correlated (r>0.6) with the score on the mHAQ and strongly negatively correlated with the physical functioning, social functioning and role limitation physical scores of the SF-36 (r<-0.6) | 0.897 with the mHAQ (n=206)  -0.774 with SF-36 physical functioning (n=206)  -0.603 with SF-36 social functioning (n=206)  -0.549 with SF-36 role limitation physical (n=198) | 3 of 4 hypotheses met. |
| Mental and emotional state | The score from the emotional and psychological well-being domain of the PMR-IS is strongly negatively correlated (r<-0.6) with the emotional well-being, social functioning and role limitation emotional scores of the SF-36. | -0.784 with emotional well-being (n=199)  -0.732 with SF-36 social functioning (n=204)  -0.610 with SF-36 role limitation emotional (n=193) | 3 of 3 hypotheses met |
| Steroid side effects | The score from the steroid side effects domain of the PMR-IS score correlates negatively (r<-0.2) with the general health scores of the SF-36. | -0.593 with general health (n=185) | Hypothesis met |
| Symptoms and function – internal relationship | The symptoms score of the PMR-IS correlates positively and moderately strongly (r>0.4) with the functional domain of the PMR-IS. | 0.834 with PMR-IS function (n=206) | Hypothesis met |

**Supplementary Table S3 – Results for responsiveness analysis**

**Mean change scores for each domain for groups defined by participants’ response to the domain-specific anchor question**

| **Domain-specific anchor response** | **Symptoms** | | **Function** | | **Psychological and emotional** | | **Steroid side effects** | |
| --- | --- | --- | --- | --- | --- | --- | --- | --- |
|  | **n** | **Mean (SD) change score** | **n** | **Mean (SD) change score** | **n** | **Mean (SD) change score** | **n** | **Mean (SD) change score** |
| **Improved** | 65 | -7.57 (20.57) | 52 | -4.60 (19.11) | 44 | -7.67 (15.18) | 29 | -3.89 (10.66) |
| **Stayed the same** | 59 | 4.22 (16.38) | 80 | 0.67 (11.99) | 95 | 1.05 (13.79) | 100 | -1.93 (13.09) |
| **Worsened** | 50 | 7.06 (11.78) | 43 | 0.88 (15.28) | 35 | -0.89 (19.18) | 23 | -1.16 (15.18) |

**Mean change scores for each domain for groups defined by participants’ response to the anchor question on overall PMR-QoL**

| **PMR-QoL anchor response** | **Symptoms** | | **Function** | | **Psychological and emotional well-being** | |
| --- | --- | --- | --- | --- | --- | --- |
|  | **n** | **Mean (SD) change score** | **n** | **Mean (SD) change score** | **n** | **Mean (SD) change score** |
| **Improved** | 51 | -6.77 (19.80) | 49 | -5.20 (17.91) | 49 | -5.61 (15.64) |
| **Stayed the same** | 74 | 2.36 (16.48) | 75 | 0.81 (11.81) | 75 | -0.08 (11.74) |
| **Worsened** | 51 | 4.69 (17.30) | 51 | 0.93 (16.62) | 50 | 0.25 (19.96) |

**Results of hypothesis testing for responsiveness**

| **Hypothesis** | **Satisfied?** | **Comments** |
| --- | --- | --- |
| There will be a qualitative trend in mean change scores on the PMR-IS symptoms domain from those reporting that their symptoms have improved a lot to those reporting that their symptoms have worsened a lot. | **Yes** | Relatively high mean change score for the group that rated themselves as staying the same.  Proportionately smaller change between the ‘worsened’ and ‘stayed the same’ groups than between the ‘improved’ and ‘stayed the same’ groups. |
| There will be a trend in mean change scores on the PMR-IS function domain from those reporting that their functional ability linked to their PMR has improved a lot to those who report that it has worsened a lot. | **Yes** | Very small difference between the ‘worsened’ and ‘stayed the same’ groups. |
| There will be a trend in mean change scores on the PMR-IS emotional and psychological well-being domain from those reporting that their psychological well-being linked to their PMR has improved a lot to those who report that it has worsened a lot. | **No** | The ‘worsened’ group showed a small improvement in their mean PMR-IS score. |
| There will be a trend in mean change scores on the PMR-IS steroid side effects domain from those reporting that their steroid side effects have improved a lot to those who report that they have worsened a lot. | **Yes** | The trend between the groups is in the expected direction but all groups showed improvement in their PMR-IS score. |
| There will be a trend in mean change scores on the symptoms, function and emotional and psychological well-being domains between those that report their overall PMR-related quality of life (PMR-QoL) as improved, the same or worsened. | **Yes** | The difference in mean change score for those in the ‘stayed the same’ as compared to the ‘worsened’ group is small, particularly for the function and emotional and psychological well-being domains. |
